# New Approach to Combat Glioblastoma Shows Promise in Preclinical Studies

**DOI:** 10.1371/journal.pmed.0030034

**Published:** 2005-12-06

Glioblastoma multiforme (GBM) develops in the tissue of the brain and grows quickly, often becoming very large before a person experiences symptoms and is diagnosed. Surgery is done to remove as much of the tumor as possible, and followed with radiation and/or chemotherapy to slow progression of the disease. But despite aggressive treatment, the cancer almost inevitably recurs, and patients usually die within a year.

Researchers are studying several ways to treat GBM using gene therapy. Some approaches target healthy cells to enhance their ability to fight cancer; others target cancer cells to destroy them or prevent their growth. Researchers are also experimenting with immune therapies, which seek to restore and stimulate the body's natural ability to recognize and attack cancer cells.

In general, scientists agree that an effective treatment for GBM must have several components. These include being highly selective to avoid damage to non-cancerous brain tissue and efficient cell killing, preferably by simultaneous activation of multiple killing mechanisms. Moreover, as it is unlikely that any treatment can target all cancer cells, it will be necessary to achieve a cancer-specific “bystander effect,” which kills neighboring tumors but not normal cells.

Alexander Levitzki and colleagues tested a therapy that tries to meet all these criteria by taking advantage of the frequent (50%–70%) overexpression of the epidermal growth factor receptors (EGFRs) in GBM. In those tumors, the number of EGFRs on tumor cells is 10–20 times higher than that on non-tumor cells. Based on this difference, they reasoned that any treatment that targets the EGFR would reach predominantly tumor cells. They built a non-viral delivery vehicle that could center in on cells expressing the EGFR and trigger internalization of the complex of the receptor and the bound vehicle. As a “toxic cargo,” Levitzki and colleagues selected double-stranded RNA (polyinosine-cytosine, poly IC). Double-stranded RNA doesn't occur naturally in eukaryotic cells, but is often associated with viral infections. To protect themselves against “viral takeover,” multicellular organisms have evolved efficient defense mechanisms that result in apoptotic death of cells that contain dsRNA. [Fig pmed-0030034-g001]

**Figure pmed-0030034-g001:**
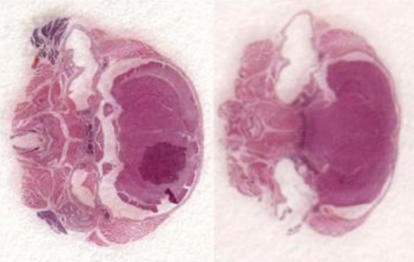
Glioblastoma in treated (right) and control (left) animals

The team found that the poly IC induced rapid apoptosis in the target cells in vitro and in glioblastomas in mice. The therapy induced complete regression of pre-established intracranial tumors in nude mice, with no obvious adverse toxic effects on normal brain tissue. And one year later, the treated mice were still disease-free and healthy.

EGFR overexpression is common in other cancer types as well, and further in vivo experiments in mice showed that non-viral delivery of poly IC completely eliminated pre-established breast cancer and adenocarcinoma xenografts derived from EGFR overexpressing cancer cell lines, suggesting that the strategy might be applicable to other cancer types. The researchers also suggest that the principle of ligand-guided delivery of dsRNA at a particular receptor that is overexpressed and undergoes endocytosis might be applicable in a range of cancers.

Experience tells that curing cancer in mice is easy and curing cancer in humans is hard. It is too early to tell whether this is one of the few approaches that will still look promising after the next round of tests. In light of the encouraging results of this study and the lack of effective treatments for GBM, however, Robert Weil (DOI: 10.1371/journal.pmed.0030031) suggests in an accompanying commentary that the use of dsRNA delivered by non-viral vectors deserves to be fast-tracked to the clinic.

